# Smelting reduction and kinetics analysis of magnetic iron in copper slag using waste cooking oil

**DOI:** 10.1038/s41598-017-02696-y

**Published:** 2017-05-25

**Authors:** Bo Li, Xubin Wang, Hua Wang, Yonggang Wei, Jianhang Hu

**Affiliations:** 1 State Key Laboratory of Complex Nonferrous Metal Resources Clean Utilization, Kunming University of Science and Technology, Kunming, 650093 China; 2 Faculty of Metallurgy and Energy Engineering, Kunming University of Science and Technology, Kunming, 650093 China

## Abstract

To improve the recovery of copper, the viscosity of copper molten slag is decreased by the reduction of magnetic iron, which, in turn, accelerates the settling and separation of copper droplets from the slag. A new technology is proposed in which waste cooking oil is used as a reductant to reduce magnetic iron in the copper smelting slag and consequently reduce carbon emissions in the copper smelting process. A kinetic model of the reduction of magnetic iron in copper slag by waste cooking oil was built using experimental data, and the accuracy of the model was verified. The results indicated that the magnetic iron content in the copper slag decreased with increasing reduction time and an increase in temperature more efficiently reduced magnetic iron in the copper slag. The magnetic iron in the copper slag gradually transformed to fayalite, and the viscosity of the copper molten slag decreased as the magnetic iron content decreased during the reduction process. The reduction of magnetic iron in the copper molten slag using waste cooking oil was a first-order reaction, and the rate-limiting step was the mass transfer of Fe_3_O_4_ through the liquid boundary layer.

## Introduction

Energy is indispensable to human beings, and the development of human society is closely related to the development and utilization of energy. Fossil fuels, including coal, oil and natural gas, have long been the primary energy source and main driving force behind industrial development and social progress. However, nonrenewable fossil fuels are a finite resource, and finding efficient, large-scale alternative energy sources is an urgent matter. The annual consumption of edible oils in China has reached tens of millions of tons. According to a waste cooking oil market analysis, the food and beverage industry generates approximately 2000 million tons of waste cooking oil in China every year, onl﻿y 8% of which is used for industrial production. Finding additional uses for waste cooking oil could result in both energy savings and emission reductions.﻿ Several groups have investigated the use of waste cooking oil as a resource for biodiesel production. Li M.^1^ studied biodiesel production from waste cooking oil using a heterogeneous catalyst from pyrolysed rice husks. In the presence of the as-prepared catalyst, the free fatty acid conversion reached 98.17% after 3 h, and the fatty acid methyl ester yield reached 87.57% after 15 h. The production of biodiesel from waste palm cooking oil using acidic ionic liquid as a catalyst was investigated by Ullah Z.^2^. The highest biodiesel yield was obtained with 5 wt.% BMIMHSO_4_, a methanol:oil ratio of 15:1, a 60 min reaction time, a temperature of 160 °C, and an agitation speed of 600 rpm, which reduced the waste cooking oil acid value to below 1.0 mg KOH/g. The final yield was 95.65 wt.%. Various studies have shown that biodiesel made from waste cooking oil can be used in diesel engines. Can Ö^3^. studied a mixture of biodiesel fuels produced from two types of waste cooking oils blended with 5% and 10% diesel fuel. The results showed that an addition of 5% and 10% biodiesel fuel resulted in a slight increase in the break specific fuel consumption (up to 4%) and a reduction in break thermal efficiency (up to 2.8%). The biodiesel additions increased the NO_x_ emissions by up to 8.7% and decreased the smoke and total hydrocarbon emissions at all engine loads. Elshaib A.A.^4^ studied the combustion characteristics, performance and exhaust emissions of a direct injection diesel engine fuelled by a diesel/biodiesel blend from waste cooking oil up to B100. The soot peak volume fraction was reduced by 15.2%, whereas the CO and HC concentrations decreased by 20 and 28.5%, respectively. The physical and chemical delay periods decreased by 1.2 and 15.8%, respectively, which resulted in an engine noise reduction of 6.5%.

Large amounts of copper slag containing significant amounts of valuable metals can be produced in the copper pyrometallurgy process. In the long term, copper slag will become a wasted resource, occupy considerable land and cause serious environmental pollution; thus, methods to further process copper slag must be developed^5^. Both chemical and physical processes contribute to copper loss in slag inclusions; however, the main reason for the loss is that strong oxidation during copper smelting can increase the magnetic iron content in the slag, which increases the viscosity and surface tension of the copper slag, causing copper slag separation to deteriorate. To reduce the copper content in the slag and improve the copper recovery, the copper slag is diluted to reduce the magnetic iron content in the slag, increase the slag mobility, and promote the settling of matte droplets^6^.

Because the copper smelting slag contains large amounts of magnetic iron, the modification of copper slag involves the reduction of iron oxides in the slag. In recent years, several studies investigating the reduction of magnetic iron in copper slag have been performed. Michal E.J.^7^ studied the effect of the CO_2_/CO ratio on the Fe^3+^/Fe^2+^ ratio by carbon in the Fe_3_O_4_ reduction process. The results showed that the magnetic iron phase increased with an increase in CO_2_ partial pressure in a reduction atmosphere and that the key factor for selective reduction by a carbonaceous reductant was control of the reducing atmosphere. Matousek J.M.^8^ summarized the effect of the reduction potential and Fe/SiO_2_ ratio on the carbon reduction process of molten copper slag at 1250 K and showed that the transformation of Fe^3+^ to Fe^2+^ in the slag requires a lower oxygen potential. The lower oxygen potential may generate metallic iron due to excessive reduction; thus, the oxygen potential should be controlled in the range of 10^−9^–10^−10^. Additionally, increasing the SiO_2_ content is more conducive to the reduction of Fe_3_O_4_. The carbon-reduced smelting of copper slag under nitrogen gas with stirring was studied by Zhang L.N.^9^. The results showed that the Fe_3_O_4_ content in the slag gradually decreased and that the main phase in the residue after the reaction included the residual magnetic iron olivine and calcium silicon solid solution. The thermodynamics and kinetics of the CH_4_ reduction of molten copper slag was studied by Riveros G. *et al*.^10^. The results showed that the reduction of Fe_3_O_4_ by CH_4_ was a first-order reaction and that the apparent activation energy of Fe_3_O_4_ reduction by CH_4_ was 62 kJ/mol, which is much lower than that when CO is used as a reducing agent^11^. Zhan H.W.^12^ studied a method of cleaning copper slag using an electric field and a slag reduction modification with mixed CH_2_ gas. These results showed that in the molten slag reduction process, the reduction rate increased with an increase in H_2_ content in the mixed gas and that the viscosity of the molten slag efficiently decreased; the phase transformation of copper oxide in the reduction process was as follows: CuFe_2_O_4_ → Cu_2_O → CuO. The interfacial interaction between the copper matte particles and the molten slag presented an opportunity to use the electrocapillary phenomenon in the slag-cleaning process. The presence of certain electric fields accelerated the migration of copper drops from the anode to the cathode and improved the copper slag cleaning. The settling rate with an electric field of 1.5 V·cm^−1^ was two or three times higher than the natural settling with only gravity.

Several traditional reducing agents, such as pulverized coal, coke, diesel and natural gas, can be used to reduce magnetic iron in copper slag in an electric furnace with the blowing and stirring method. The main elements of waste cooking oil are C, H, and O; thus, the chemical composition of waste cooking oil, which is a green resource, is notably similar to that of petroleum diesel. It is highly significant that a waste resource, such as waste cooking oil, can be used in a copper slag depletion process thereby reducing carbon emissions.

## Test Materials and Methods

### Test materials

The waste cooking oil used in this study was obtained from the waste oil and fats from the food and beverage industry in Kunming. The chemical compositions are shown in Table 1. The primary elements of the waste cooking oil were C, H, and O with a small quantity of N and S. C and H accounted for 90% of the total waste cooking oil, and the O content was also notable. In the high-temperature pyrolysis process, high temperature was more beneficial to the gasification and generation of gas-phase products. The copper slag used in this study was the electric furnace slag from the Yunnan copper factory; its chemical composition is shown in Table 2.

**Table 1:** Elemental composition of waste cooking oil.

Element	C	H	O	N	S
Content (wt.%)	77.81	12.33	8.93	0.86	0.07

**Table 2:** Chemical composition of copper slag.

Element	SiO_2_	Fe_3_O_4_	CaO	Al_2_O_3_	MgO	S	Cu
Content (wt.%)	34.36	17.80	6.44	5.29	4.38	0.85	0.58

### Test apparatus and method

**Figure 1:**
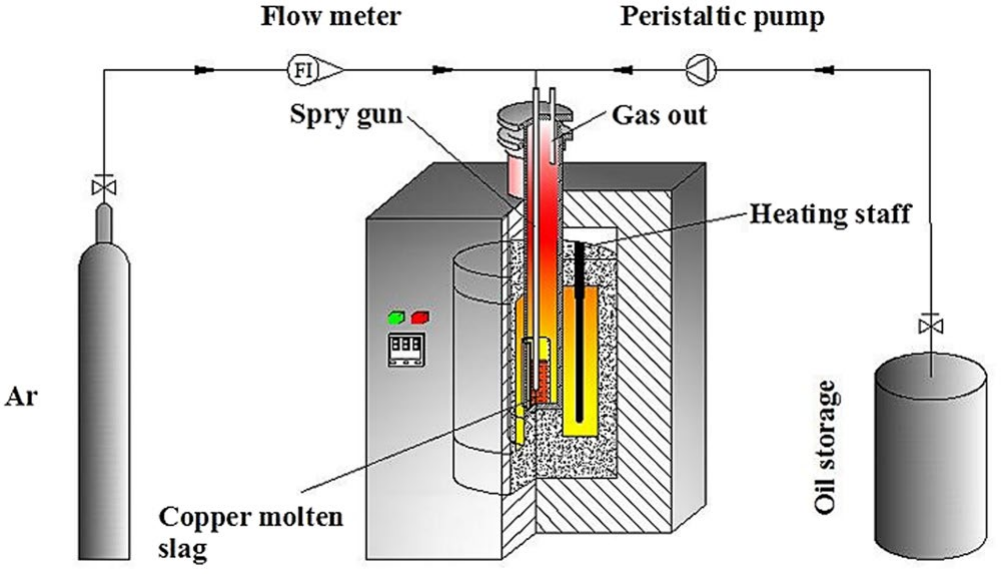
The apparatus of magnetic iron reduction by waste cooking oil.

The apparatus for reducing magnetic iron using waste cooking oil is shown in Fig. 1. The main component of the equipment is an electric tube furnace. A crucible with copper slag was placed in the furnace tube, and the top of the furnace tube was sealed using a stainless steel flange seal. First, argon gas was bubbled into the furnace tube to remove any remaining air in the device before the experiment. Next, the furnace temperature was increased to the experimental temperature and maintained for 40 min. After the waste cooking oil was preheated to 50 °C, it was blown into the copper slag sample using a peristaltic pump with argon as the carrier gas. The oil flow was 1 mL/min, and the argon flow was 3 L/min during the experiment. To sufficiently reduce and deposit copper matte droplets in the slag, the temperature was maintained for 30 min after the end of the experiment. Highly pure argon gas at a flow rate of 600 mL/min was used as a protective gas in the cooling process.

The elemental composition of C, H and N in the waste cooking oil was determined using a Perkin Elmer 2400 Series II element analyser, the S content was determined with a TS-2000 sulfur content analyser, and the O content was obtained in a difference calculation. X-ray diffraction techniques were used to measure the phase changes using a Cu-Kα radiation source (35 kV, 20 mA) to obtain data at a scanning rate of 8°/min over the angular range of 10° to 90°. A scanning electron microscopy analysis of the concentrates was performed using a HITACHI-S3400N SEM with a BSE resolution of 4.0 nm (30 kV). An energy dispersive spectroscopy analysis was performed using EDAX. The magnetic iron content of the experimental product was analysed using a Satmagan135 magnetic analyser.

## Discussion

### Change in the magnetic iron content in the copper slag during the reduction process

**Figure 2:**
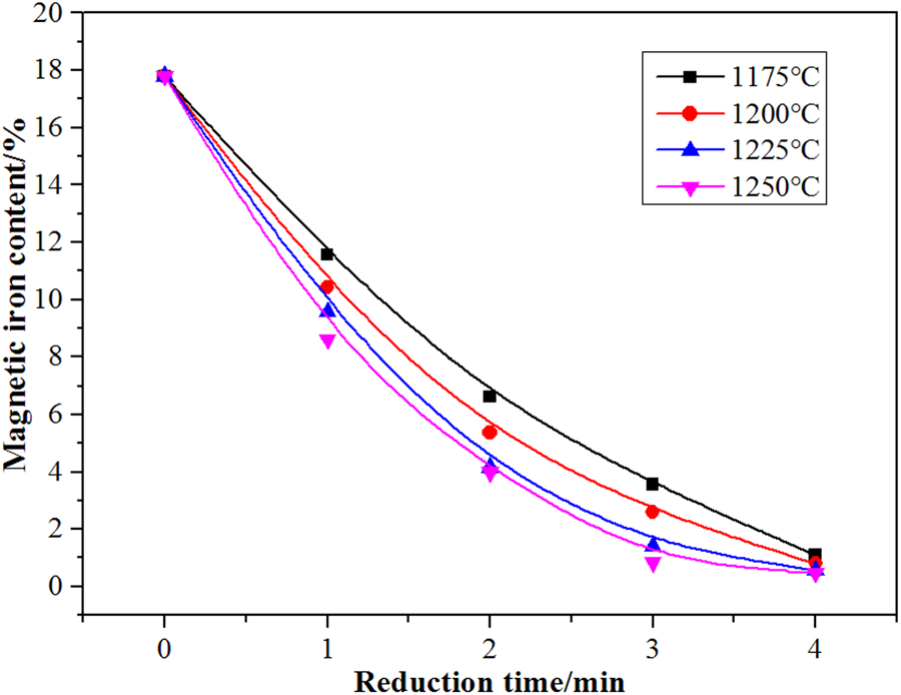
The change of content of magnetic iron with reduction time at different temperatures.

**Figure 3:**
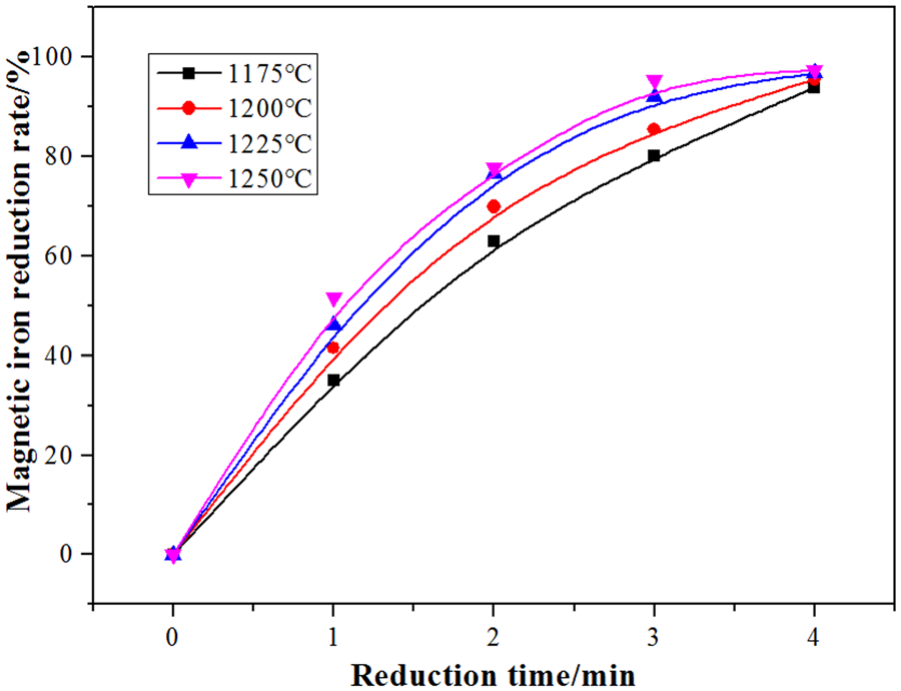
The change of reduction rate of magnetic iron with reduction time at different temperatures.

Figure 2 and Fig. 3 show the changes in the content and reduction rate of magnetic iron with respect to the reduction reaction time at different temperatures. When the reduction time reached 4 min, the magnetic iron content in the slag decreased to below 2% and the reduction rate of magnetic iron was greater than 90%. These results showed that the magnetic iron in the copper slag was significantly reduced using waste oil. As the temperature increased, the reduction rate of the magnetic iron in the copper slag slightly increased. Because the viscosity of the copper slag decreased with increasing temperature, the dynamic conditions of reduction were improved.

### Analysis of the phase and viscosity of the copper slag in the reduction process

**Figure 4:**
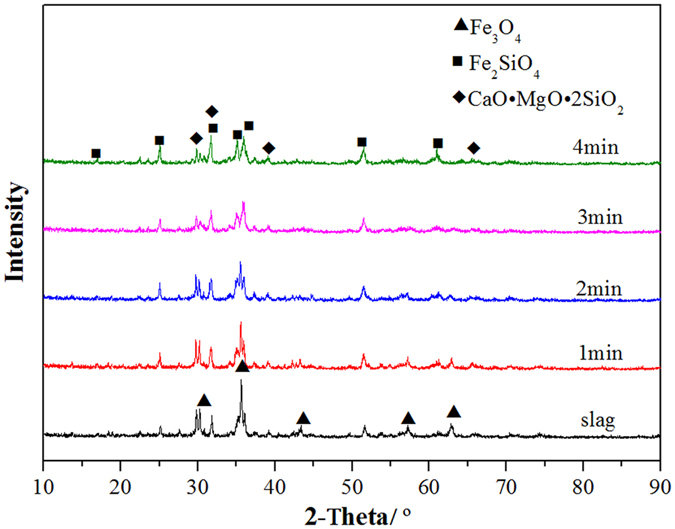
XRD patterns of copper slag for different times (1175 °C).

The XRD patterns of the copper slag that was reduced using waste oil at 1175 °C for different reduction times are shown in Fig. 4. Magnetic iron (Fe_3_O_4_), the main phase in electric furnace slag, was transformed into fayalite (Fe_2_SiO_4_) and a silicate solid solution, which included calcium magnesium oxide. As the reduction time increased, the peak intensity of the magnetic iron in the copper slag gradually decreased and the peak intensity of fayalite gradually increased. The results show that the magnetic iron in the copper slag is transformed into fayalite in the reduction process. The magnetic iron in the slag is reduced to FeO, which generates fayalite and SiO_2_.

**Figure 5:**
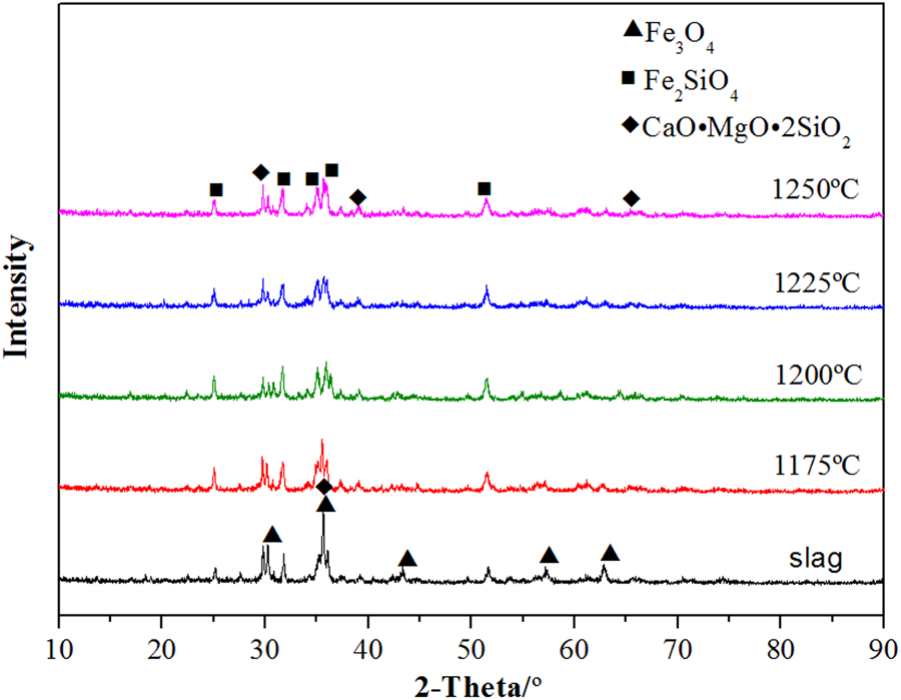
XRD patterns of copper slag for different temperatures (2 min).

Figure 5 shows the XRD patterns of the copper slag reduced at different temperatures for 2 min. Compared with the original slag, the peak intensities of the magnetic iron in the reduced copper slag are much weaker than those under other conditions. As the temperature increased, the peak intensity of the magnetic iron in the copper slag gradually decreased, and the peak intensity of fayalite gradually increased. Compared with the reduction time, the temperature has a smaller effect on the phase of the copper slag.

**Figure 6:**
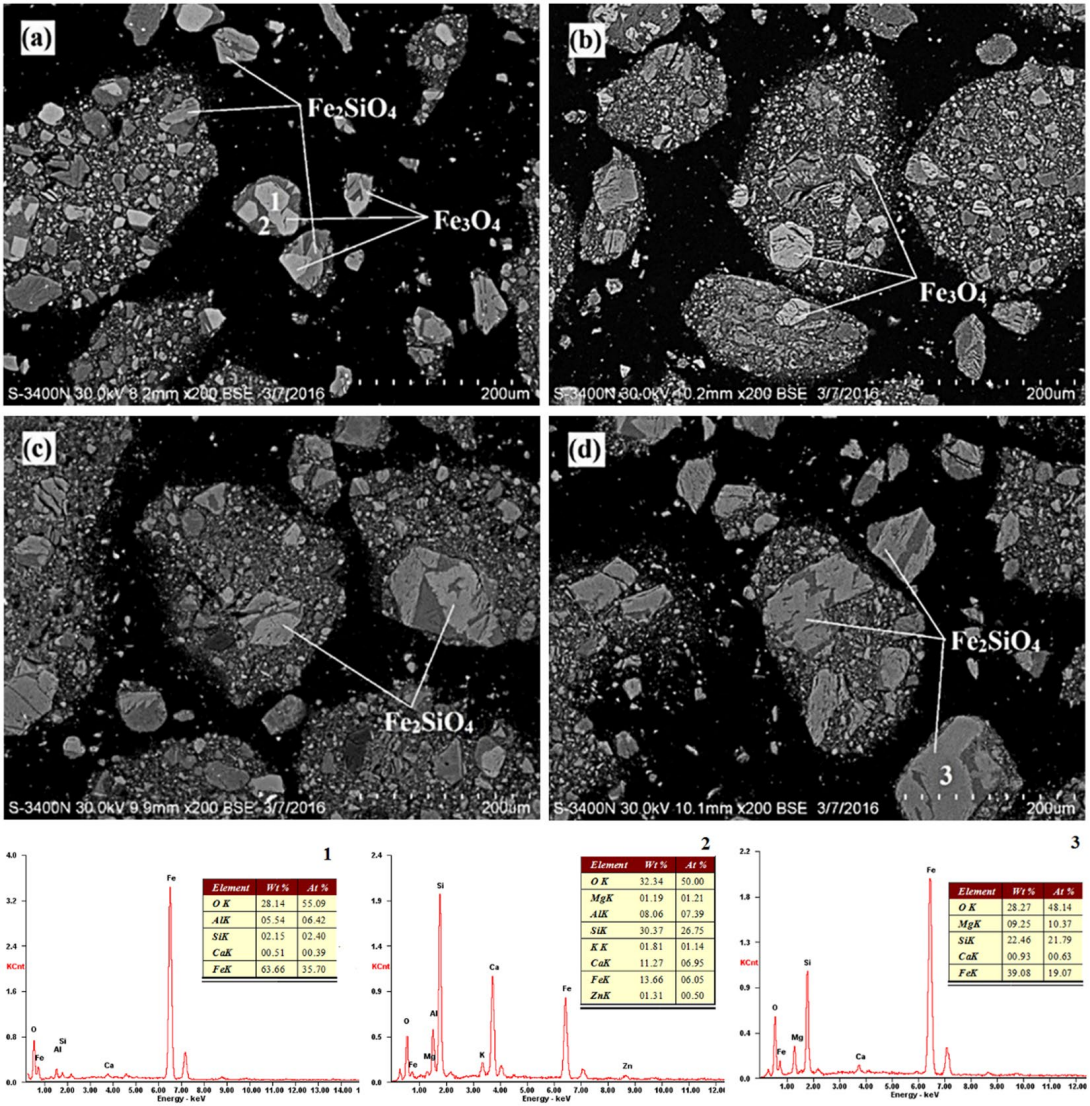
The SEM patterns of copper slag for different reduction times (1175 °C): (**a**) 1 min, (**b**) 2 min, (**c**) 3 min, (**d**) 4 min.

The SEM patterns of the copper slag that was reduced at 1175 °C for various durations are shown in Fig. 6. With an increase in reduction time, the magnetic iron regions decreased in intensity, and the fayalite regions gradually increased. Thus, the magnetic iron phase was converted to fayalite with the reduction of the copper slag.

**Figure 7:**
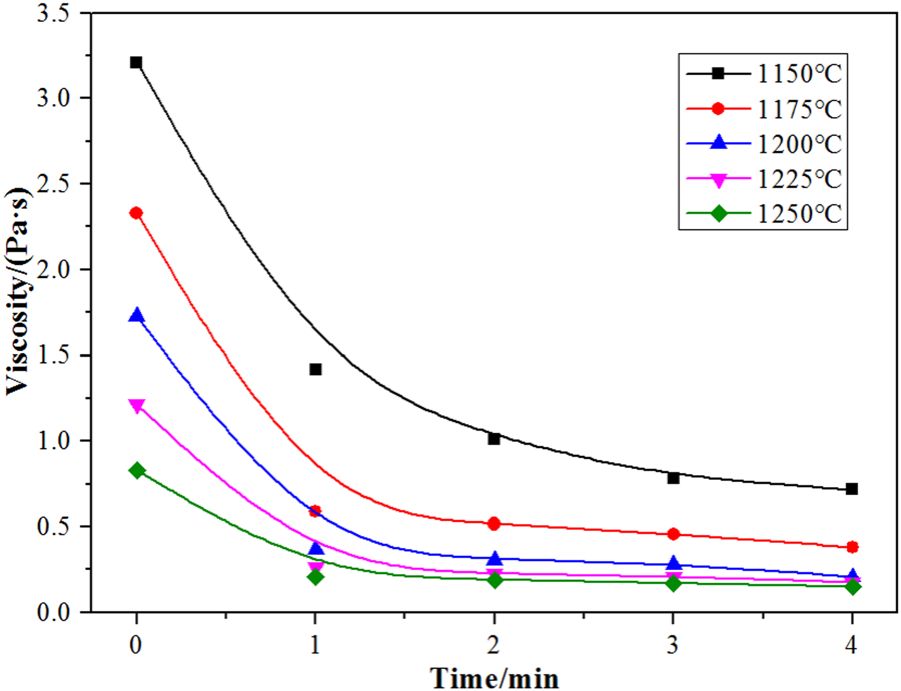
The relationship between the viscosity of copper slag and the reduction time.

**Figure 8:**
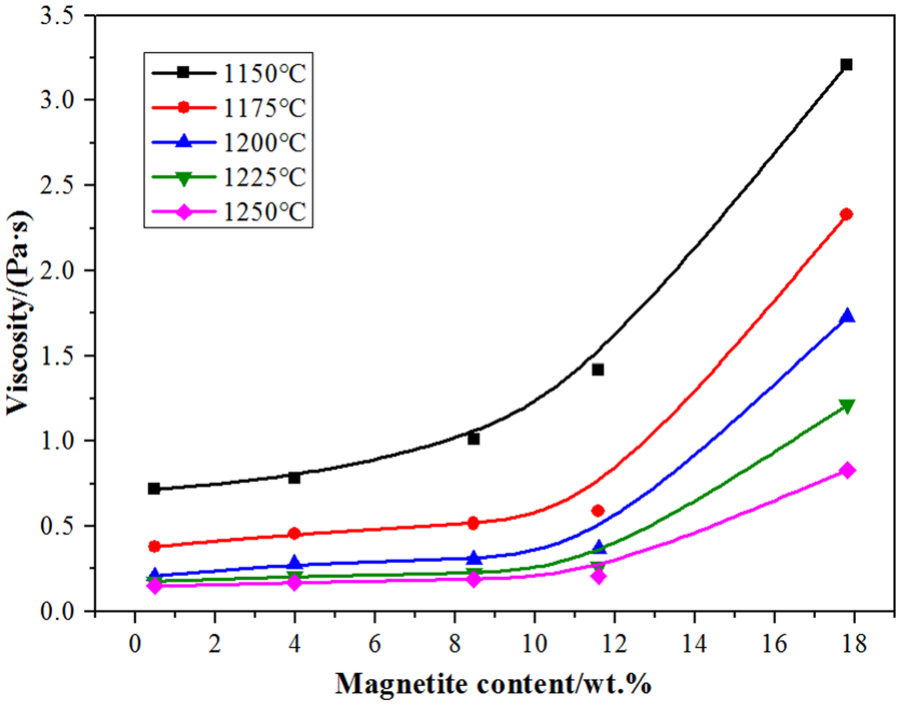
The relationship between the variety of Fe_3_O_4_ content in slag and the viscosity of copper slag.

The waste cooking oil decreased the slag viscosity by reducing the magnetic iron content in the copper slag. Decreasing the slag viscosity can improve the kinetics of the separation of copper slag droplets. The viscosity of the sample was determined at a reduction temperature of 1175 °C for different reduction times. The viscosity curves of the samples at different temperatures are shown in Fig. 7. The viscosity of the copper slag decreases with an increase in reduction time. Generally, the copper slag viscosity is less than 0.5 Pa∙s, and the copper slag can be considered to have good liquidity; later, the viscosity increases to greater than 1 Pa∙s, which is considered poor liquidity^13^. When the reduction time is 3 min, the copper slag viscosity decreases to 1 Pa∙s. The relationship between the magnetic iron content and the viscosity of the copper slag at different reduction temperatures is shown in Fig. 8. When the magnetic iron content in the copper slag is below 10%, the slag viscosity does not change significantly. However, when the magnetic iron content is above 10%, the slag viscosity increases sharply with increasing magnetic iron content.

### Kinetics analysis of the reduction of magnetic iron

**Figure 9:**
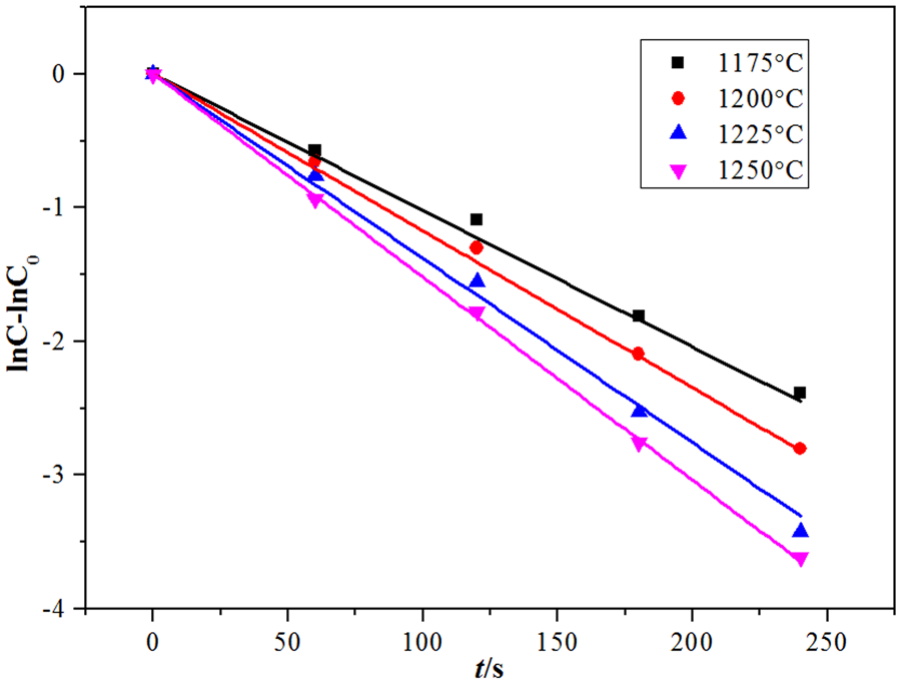
The relationship between the concentration of Fe_3_O_4_ in copper slag and reaction time (n = 1).

**Figure 10:**
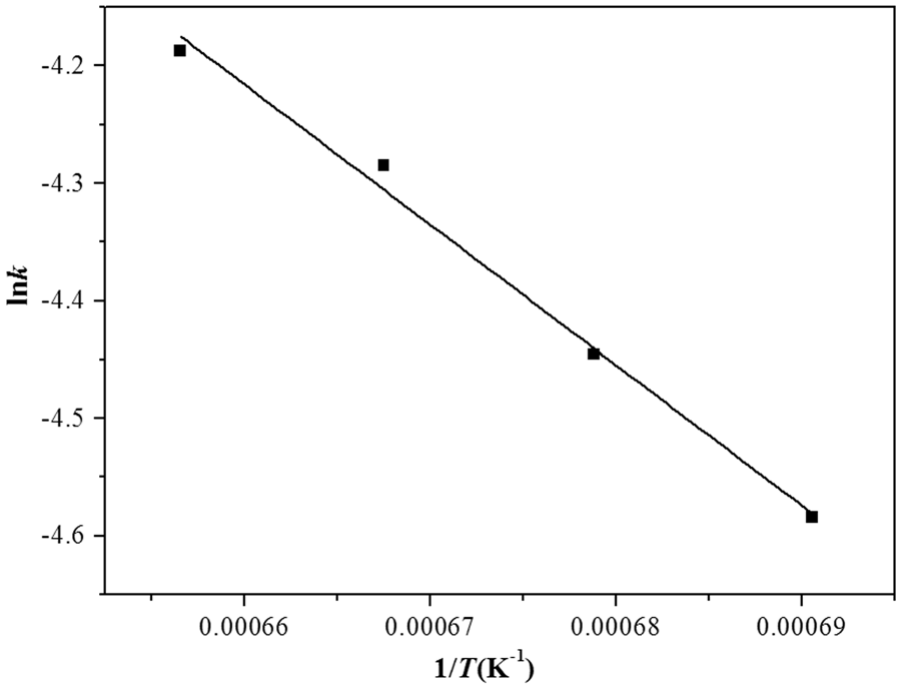
The relationship between lnK and 1/T.

The relationship between the magnetic iron concentration in the copper slag and the reaction time of the copper slag reduction by waste cooking oil is shown in Fig. 9. Because a linear correlation between the magnetic iron concentration and the reaction time is better for the reaction order (n = 1), the reduction of magnetic iron in the copper slag is a first-order reaction^14^. According to gas-liquid reaction kinetics, when the logarithm of the concentration of the diffusion components in the liquid phase is linearly related to the reaction time, the gas-liquid reaction is controlled by the liquid phase mass transfer. Thus, the reduction of magnetic iron in the copper slag by waste cooking oil is controlled by liquid phase mass transfer^15, 16^. Figure 10 shows the relationship between lnK and 1/T at different temperatures. Because the linearity is notably good, the apparent activation energy and frequency factor, which were obtained from the intercept and slope of a fitted straight line, are shown in Table 3. The apparent activation energy is 99.3 kJ/mol, and the pre-exponential factor is 39.2.

**Table 3:** Kinetic parameters of magnetic iron in the reduction of copper slag.

T/°C	*k*/s^−1^	*n*	*E*/kJ·mol^−1^	*A*/s^−1^
1175	0.01021	1	99.3	39.2
1200	0.01173			
1225	0.01378			
1250	0.01519			

**Figure 11:**
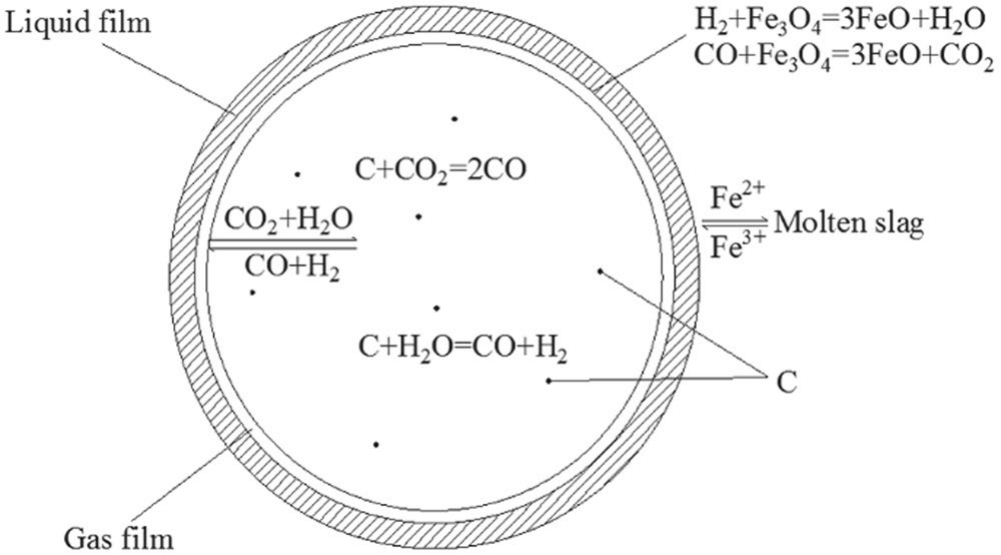
The reaction model of magnetic iron in the reduction process of copper slag by waste cooking oil.

The pyrolysis of waste cooking oil rapidly forms small gaseous molecules and coke at high temperature; thus, the reduction of magnetic iron in copper slag is a gas-liquid-solid phase reaction. Following the double-film theory, the reaction model of magnetic iron reduction in copper slag by waste cooking oil is shown in Fig. 11.

When bubbles come into contact with the slag, a stable phase interface is formed on both layers of the vapour film and the layer of the liquid film. The resistance to mass transfer mainly occurs in the two-layer films. The reducing gas diffuses from the body of the gas phase to the gas-liquid phase interface in the bubbles. Fe_3_O_4_ diffuses from the body of the liquid slag to the gas-liquid phase interface. The reaction generates reductants, such as FeO, CO_2_, and H_2_O, at the gas-liquid phase interface, and FeO diffuses into the slag body. The gas products diffuse into the body of the gas phase, which may continue to react with coke to generate CO and H_2_^17–19^.

The reduction of magnetic iron by waste cooking oil is controlled by the liquid phase:

1 $$k={k}_{{{\rm{Fe}}}_{{\rm{3}}}{{\rm{O}}}_{{\rm{4}}}}$$

The mass transfer flux of Fe_3_O_4_ through a liquid membrane^20^ can be represented as follows:

2 $${J}_{{{\rm{Fe}}}_{{\rm{3}}}{{\rm{O}}}_{{\rm{4}}}}={k}_{{{\rm{Fe}}}_{{\rm{3}}}{{\rm{O}}}_{{\rm{4}}}}({C}_{{{\rm{Fe}}}_{{\rm{3}}}{{\rm{O}}}_{{\rm{4}}}}-{{C}^{\ast }}_{{{\rm{Fe}}}_{{\rm{3}}}{{\rm{O}}}_{{\rm{4}}}})$$

where ${J}_{{{\rm{Fe}}}_{{\rm{3}}}{{\rm{O}}}_{{\rm{4}}}}$ is the mass transfer flux, mol/(m^2^∙s); ${k}_{{{\rm{Fe}}}_{{\rm{3}}}{{\rm{O}}}_{{\rm{4}}}}$ is the Fe_3_O_4_ mass transfer coefficient, m/s; ${C}_{{{\rm{Fe}}}_{{\rm{3}}}{{\rm{O}}}_{{\rm{4}}}}$ is the Fe_3_O_4_ concentration in the slag body, mol/m^3^; and ${{C}^{\ast }}_{{{\rm{Fe}}}_{{\rm{3}}}{{\rm{O}}}_{{\rm{4}}}}$ is the Fe_3_O_4_ concentration at the gas-liquid interface, mol/m^3^.

According to the film-penetration theory^21^, the mass transfer coefficient can be expressed as:

3 $${k}_{{{\rm{Fe}}}_{{\rm{3}}}{{\rm{O}}}_{{\rm{4}}}}=2\sqrt{\frac{{D}_{{{\rm{Fe}}}_{{\rm{3}}}{{\rm{O}}}_{{\rm{4}}}}}{\pi {t}_{c}}}$$

where ${D}_{{{\rm{Fe}}}_{{\rm{3}}}{{\rm{O}}}_{{\rm{4}}}}$ is the diffusion coefficient of Fe_3_O_4_ in the liquid film, m^2^/s, and *t*_*c*_ is the mass transfer time. From this, the following formula can be obtained:

4 $${t}_{c}={d}_{e}/{u}_{b}$$

when the gas flow at the nozzle exit is small, the size of the bubble is determined by the balance between the buoyancy and the surface tension^21^:

5 $$\frac{\pi }{6}{{d}_{e}}^{3}({\rho }_{l}-{\rho }_{b})g=\pi {d}_{0}\sigma $$

where ${d}_{e}$ is the bubble diameter, m; ${\rho }_{l}$ is the slag density, kg/m^3^; ${\rho }_{b}$ is the bubble density, kg/m^3^; $\sigma$ is the surface tension of the slag, N/m; and ${d}_{0}$ is the nozzle inner diameter, m.

The floatation speed of a bubble in the slag is calculated using the Stokes formula^12^:

6 $${u}_{b}=\frac{{{d}_{e}}^{2}}{12{\mu }_{l}}({\rho }_{l}-{\rho }_{g})$$

where ${\mu }_{l}$ is the slag viscosity, Pa∙s.

The mass transfer coefficient between the slag and the bubble is related to the bubble movement and the diffusion phase. Based on experimental data, the semi-empirical and semi-theoretical formulae were fitted and the criterion numbers are as follows^22, 23^:

Reynolds number:

7 $${\rm{Re}}=\frac{{\rho }_{l}{u}_{b}{d}_{e}}{{\mu }_{l}}$$

Sherwood standard:

8 $$Sh=\frac{{k}_{{\rm{d}}}{d}_{{\rm{e}}}}{D}$$

Schmidt standard:

9 $$Sc=\frac{{\mu }_{l}}{{\rho }_{l}D}$$

The mass transfer coefficient between the bubbles and the slag can be expressed as:

10 $$Sh=mR{e}^{n}S{c}^{l}$$

The parameters related to the kinetics analysis at different temperatures are shown in Tables 4–7. A nonlinear fit of the multi-variates and multi-parameters provides the undetermined parameters^24^.

11 $$Sh=4.13\times {10}^{-3}\cdot R{e}^{1.18}\cdot S{c}^{1.82}$$

**Table 4:** Physical parameters of slag under different temperatures.

Temperature *T*/°C	Density ${\rho }_{l}$/kg∙m^−3^	Viscosity ${\mu }_{l}$/Pa∙s	surface tension $\sigma$/10^−3^ N∙m^−1^
1175	3566	2.33	575.28
1200	3438	1.73	605.45
1225	3351	1.21	635.62
1250	3306	0.83	665.78

**Table 5:** Pyrolysis of waste oil in different temperature.

Temperature *T*/°C	H_2_ productivity /mL∙min^−1^	CO productivity /mL∙min^−1^	Total productivity /mL∙min^−1^	Coke productivity /g∙min^−1^
1175	776	97	873	0.51
1200	819	105	924	0.52
1225	838	112	951	0.53
1250	858	120	978	0.54

**Table 6:** Bubble characteristic parameters at different temperatures.

Temperature *T* /°C	Density ${\rho }_{b}$/kg∙m^−3^	Diameter *d*_e_ /m	Residence time *t*_e_/s	Diffusion coefficient ${D}_{{{\rm{Fe}}}_{{\rm{3}}}{{\rm{O}}}_{{\rm{4}}}}$/m^2^∙s^−1^	Floating velocity *u*_b_/m∙s^−1^
1175	0.287	8.40 × 10^−3^	0.113	9.16 × 10^−6^	0.074
1200	0.280	8.71 × 10^−3^	0.086	9.56 × 10^−6^	0.100
1225	0.274	8.87 × 10^−3^	0.066	9.51 × 10^−6^	0.135
1250	0.268	9.04 × 10^−3^	0.051	9.36 × 10^−6^	0.177

**Table 7:** Bubble motion criterion at different temperatures.

Temperature *T* /°C	Re	Sc	Sh
1175	0.95	71.3	9.36
1200	1.73	52.6	10.69
1225	3.32	38.0	12.85
1250	6.37	26.8	14.67

The reduction rate of magnetic iron can be expressed as:

12 $$R=(1-{e}^{-4.13\times {10}^{-3}\cdot \frac{{{u}_{{\rm{b}}}}^{1.18}{{\mu }_{{\rm{l}}}}^{0.64}{{d}_{{\rm{e}}}}^{0.18}}{{{\rho }_{{\rm{l}}}}^{0.64}{D}_{{{\rm{Fe}}}_{{\rm{3}}}{{\rm{O}}}_{{\rm{4}}}}^{0.82}}t})\times 100 \% $$

**Figure 12:**
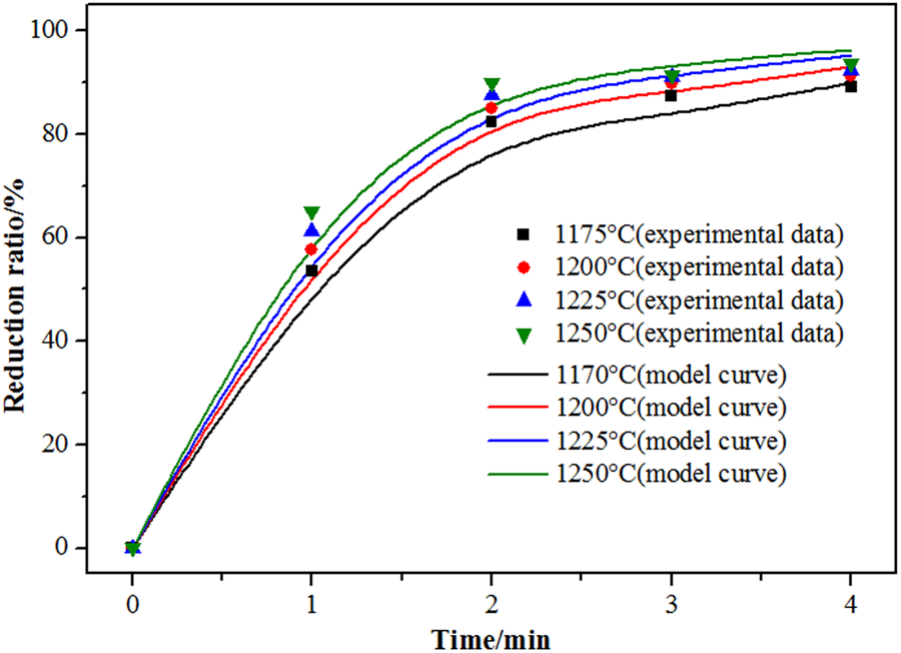
Comparison of numerical and experimental data (2 mL/min).

To validate the accuracy of the model, the flow of waste cooking oil in the experimental process was set at 2 mL/min. The experimental data for the reduction rate of magnetic iron at different temperatures and the calculated data based on the above model are shown in Fig. 12. The results show that the experimental data are consistent with the calculated results.

## Conclusions

Waste cooking oil is a green resource. It is highly significant that waste cooking oil is used in a copper slag depletion process to achieve waste resource utilization and reduce carbon emissions. In this study, electric furnace copper slag was found to be composed of magnetic iron (Fe_3_O_4_), fayalite (Fe_2_SiO_4_), calcium magnesium oxide, and a silicate solid solution. The magnetic iron content in the copper slag decreased with an increase in reduction time at different temperatures. With a reduction time of 4 min, the magnetic iron content in the copper slag was reduced to less than 2%, and the reduction efficiency of magnetic iron exceeded 90%.

When the magnetic iron content in the copper slag was below 10%, the slag viscosity did not noticeably change. However, when the magnetic iron content was above 10%, the slag viscosity increased sharply with increasing magnetic iron content.

The reduction of magnetic iron in the copper slag was shown to be a first-order reaction, and the apparent activation energy was 99.3 kJ/mol. The reduction of magnetic iron was controlled by liquid phase mass transfer, and the reduction rate of magnetic iron can be expressed as:

$$R=(1-{e}^{-4.13\times {10}^{-3}\cdot \frac{{{u}_{{\rm{b}}}}^{1.18}{{\mu }_{{\rm{l}}}}^{0.64}{{d}_{{\rm{e}}}}^{0.18}}{{{\rho }_{{\rm{l}}}}^{0.64}{D}_{{{\rm{Fe}}}_{{\rm{3}}}{{\rm{O}}}_{{\rm{4}}}}^{0.82}}t})\times 100 \% $$
